# Nitrogen- and Oxygen-Containing Three-Dimensional Hierarchical Porous Graphitic Carbon for Advanced Supercapacitor

**DOI:** 10.3390/nano10081540

**Published:** 2020-08-06

**Authors:** Yunyan Zhao, Honghu Wang, Jing Liu, Jinghao Liu, Guicun Li, Hongrui Peng, Kezheng Chen, Zhonghua Zhang

**Affiliations:** College of Materials Science and Engineering, Qingdao University of Science and Technology, Qingdao 266042, China; zhaoyy@qust.edu.cn (Y.Z.); tzswhh@126.com (H.W.); jingliu@qust.edu.cn (J.L.); liujh@glabat.com (J.L.); penghongrui@qust.edu.cn (H.P.); kchen@qust.edu.cn (K.C.)

**Keywords:** hierarchical porous structures, porous graphitic carbon, polypyrrole, supercapacitors, Ni catalyst

## Abstract

Three-dimensional hierarchical porous graphitic carbon (HPGC) were synthesized via one-step carbonization-activation and a catalytic strategy. The method can not only improve the graphitization degree of carbon materials, but also offer plentiful interfaces for charge accumulation and short paths for ion/electron transport. Polypyrrole, potassium hydroxide, and nickel acetate were used as the carbon precursors, activating agent, and catalyst, respectively. The retraction and dissolution of Ni caused the change of pore size in the material and led to the interconnected micro/nano holes. Nickel acetate played a significant role in enhancing the electrical conductivity, introducing pseudocapacitance, and promoting ion diffusion. In the supercapacitor, HPGC electrode exhibited a remarkable specific capacitance of 336.3 F g^−1^ under 0.5 A g^−1^ current density and showed high rate capability, even with large current densities applied (up to 50 A g^−1^). Moreover, HPGC showed optimal cycling stability with 97.4% capacitance retention followed by 3000 charge-discharge cycles. The excellent electrochemical performances coupled with a facile large-scale synthesis procedure make HPGC a promising alternative for supercapacitors.

## 1. Introduction

As a novel type of energy-storage device, supercapacitors have recently received considerable attention since they are essential components of high-rate electric devices in hybrid vehicles [1,2,3,4]. Activated carbons with a large specific surface area (SSA) over 2000 m^2^ g^−1^, one type of electrode materials for supercapacitors, have achieved commercial success in supercapacitor devices due to their low cost [5,6,7]. The mechanism of energy storage is based on the accumulation of charge in the electric double layer formed at the electrode/electrolyte interface [8,9]. It is reported that some nitrogen- and oxygen-containing functional groups play important roles in enhancing the electrochemical performance of the carbon-based materials due to the introduction of pseudocapacitance and improving electrolyte wettability [10,11,12]. Compared with heteroatoms doping in carbon-based materials, using heteroatoms-containing polymers as precursors is an easier way to obtain nitrogen- and oxygen-containing carbon [13]. Heteroatoms-containing polymers mainly contain biomass, natural polymers and synthetic polymers [14]. Among them, synthetic polymer precursors are very popular because the surface/interface properties and chemical structure of the precursors can be tailored and designed. Polypyrrole (PPy) has great potential for preparing carbon-based materials due to the simple synthetic method, low cost, large specific surface area, good processability, and chemical stability.

However, there still exist several disadvantages for carbon-based materials, such as the sluggish ion diffusion and low electrical conductivity [15,16,17]. Sluggish ion diffusion may cause capacity loss and low power density during the fast charging/discharging process [18]. In recent years, some studies have illustrated that hierarchical porous carbon (HPC) has a high specific surface area and interconnected pores structure to guarantee the rapid electrolyte ions transfer within the electrode materials [19,20]. Typically, HPC can be obtained by either physical activation with different oxidizing atmospheres or chemical activation with KOH, H_3_PO_4_, or ZnCl_2_ [20]. According to the literature, oxygen- and nitrogen-enriched porous carbon materials showed high specific capacitance with excellent cycling stability [20,21,22].

Nevertheless, the low electron conductivity of the above carbon materials limits their rate capability and energy efficiency. The electrical conductivity of carbon-based materials can be effectively improved by graphitization [23]. Generally, high-temperature or/and high-pressure treatment of carbon precursor and catalytic graphitization are major methods to improve the degree of graphization [24]. Catalytic graphitization is a better option, to avoid the vast collapse of pore structure [25]. Even so, a high graphitization degree may lead to an undeveloped pore structure and the reduction of the specific surface area, and further lead to the sluggish ion diffusion [26]. However, our research found that the catalyst caused the collapse of macropores (>50 nm) and micropores (<2 nm), but created more mesopores (from 2 to 50 nm) and the interconnection of pores.

Herein, we report a facile large-scale synthesis method of activated carbon, which can not only improve the graphitization degree, but also offer plentiful interfaces for charge accumulation and short paths for ion/electron transport. Novel functionalized hierarchical porous graphitic carbon (HPGC) with interconnected micro/nano holes was synthesized via simultaneous activation of KOH and catalysis of Ni (Ac)_2_·4H_2_O using PPy as the precursor. The presence of nickel catalyst not only increases the graphitization degree of the material, but also creates more interconnected pores. Hence, our HPGC displayed excellent electrochemical performance and a high lifetime cycling stability.

## 2. Experimental

### 2.1. Synthesis of Hierarchical Porous Graphitic Carbon (HPGC) Materials

**Figure 1:**
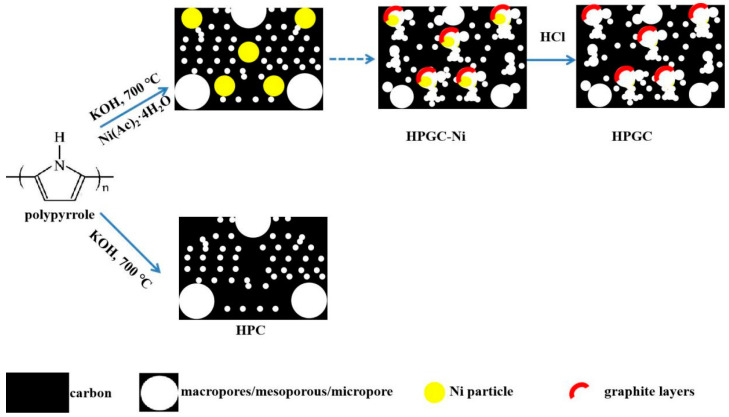
Schematic showing the preparation of hierarchical porous carbon (HPC) and hierarchical porous graphitic carbon (HPGC).

The PPy was obtained in a chemical polymerization step using a templateless procedure [27,28]. In a typical synthesis, 2 mL of pyrrole monomer was dissolved in 290 mL of 0.1 mol L^−1^ HCl solution under ultrasonication for 15 min, and then 50 mL of (NH_4_)_2_S_2_O_8_ (6.8 g) aqueous solution was added to the above solution. The molar ratio of pyrrole monomer, doping agent (HCl), and oxidizing agent (ammonium persulfate) was 1:1:1. The mixture was magnetically stirred for 5 min. After that, the polymerization reaction occurred at low temperature (0–5 °C) for 24 h without any disturbance. The formed PPy products were filtered and washed with 3 wt.% ammonia water solution, deionized water, and ethanol several times until the filtrate became neutral. The as-prepared PPy products (2 g) and Ni (Ac)_2_·4H_2_O (1.44g) were dispersed in 50 mL of 25 *v*/*v*% ethanol/deionized water solution by stirring at room temperature for 10 h. Subsequently, 6 g of KOH was added into the mixture under stirring. Then, the solvent was removed via vacuum evaporation at 50 °C. The obtained powder was carbonized in a tubular furnace under nitrogen atmosphere at 700 °C for 2 h with a heating rate of 3 °C min^−1^. After carbonization, the inorganic impurities were removed with enough deionized water to obtain hierarchical porous graphitic carbon@ nickel (HPGC-Ni) composite (Figure 1). Finally, HPGC-Ni composite was etched in 1 mol L^−1^ of HCl solution at 80 °C for 24 h to remove Ni and impurities (residual KOH), washed with deionized water, and then dried at 80 °C in an oven for 12 h to obtain HPGC. For comparison, HPC was obtained under the same conditions without Ni (Ac)_2_·4H_2_O. Analytical-grade pyrrole was purchased from Aldrich (Shanghai, China). Other chemical reagents were purchased from Sanhe (Yantai, China) and used as received.

### 2.2. Materials Characterization

The morphology of the samples was investigated by transmission electron microscopy (TEM, FEI Tecnai G20, (FEI Ltd., Hillsboro, OR, USA)) and field-emission scanning electron microscopy (SEM, JSM6700F, JEOL Ltd., Tokyo, Japan). Energy-dispersive spectrometer (EDS) mapping images were also carried out on the JSM6700F electron microscope (JEOL Ltd., Tokyo, Japan) operated at 20 kV. The high-resolution transmission electron microscopy (HRTEM) images were carried out on a JEOL JEM-2010 electron microscope (JEOL Ltd., Tokyo, Japan) operated at 200 kV. X-ray diffraction (XRD) analysis was conducted with a Rigaku D-max-X-γA diffractometer (Rigaku Ltd., Tokyo, Japan) and Cu Kα radiation (λ = 1.54178 Å). Nitrogen adsorption-desorption isotherms were measured on a micrometrics ASAP 2020 analyzer (Micromeritics Instrument corp., Atlanta, GA, USA) at −196 °C. Before measurements, the samples were degassed at 150 °C under vacuum for 4 h. A nitrogen adsorption/desorption test (Micromeritics ASAP2020) was used for obtaining the overall specific surface area by the Brunauer–Emmett–Teller (BET) theory. The total pore volume, calculated from the adsorbed amount at a relative pressure of 0.99, was determined by the Barrett–Joyner–Halenda (BJH) method. X-ray photoelectron spectroscopy (XPS) experiments were performed at a Perkin–Elmer PHI 550 spectrometer (Perkin-Elmer corp., Waltham, MA, USA). The XPS spectra were recorded using monochromatized radiation energy of 1486.6 eV. Deconvolution of XPS peaks was performed by a CasaXPS software (version 2.3.13, Casa Software Ltd., Beijing, China). Fourier transform infrared spectroscopy (FTIR) was obtained with a CARY500UV-VIS-NI spectrometer (Cary Ltd., Palo Alto, CA, USA) in a range from 4000 cm^−1^ to 400 cm^−1^.

### 2.3. Electrochemical Measurements

The electrochemical performance of the samples was measured via a three-electrode system in a 1 mol L^−1^ of H_2_SO_4_ electrolyte solution [17,29]. The Pt foil and saturated calomel electrode (SCE) were taken as counter electrode and reference electrode, respectively. The reference voltage was 0.245 V. All electrochemical tests were assessed on an Autolab Potentiostat/Galvanostat, PGSTAT302N. The working electrodes were prepared by mixing activated carbon (75 wt%), acetylene black (20 wt%), and polytetraflfluoroethylene binder (5 wt%) in ethanol solvent [30]. The slurry was rolled on a stainless steel (1 × 1 cm) current collector and dried overnight at 80 °C. The as-prepared electrodes were pressed under a pressure of 10 MPa for 60 s and then dried at 80 °C for 24 h. Cyclic voltammetry (CV) was performed in a potential range from −0.2 V to 0.8 V by varying the scan rate from 5 to 100 mV s^−1^. Galvanostatic charge-discharge measurements were performed at various current densities of 0.5–50 A g^−1^. For the Nyquist plots were obtained in the frequency range of 10 KHz to 10 MHz at open circuit potential. Polytetraflfluoroethylene was purchased from Aldrich (Shanghai, China). Other chemical reagents were purchased from Sanhe (Yantai, China) and used as received.

## 3. Results and Discussion

**Figure 2:**
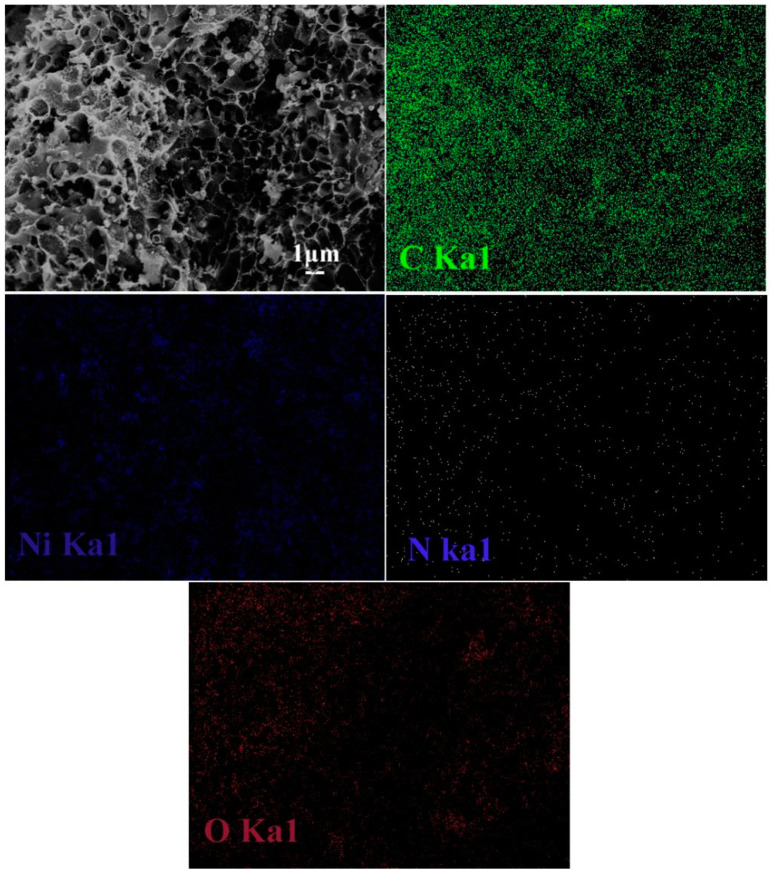
Scanning electron microscopy (SEM) image of HPGC-Ni and the corresponding energy-dispersive spectrometer (EDS) mapping images.

The uniform distribution of Ni element in HPGC-Ni is critical to the electrical conductivity of HPGC. The position of Ni catalyst in HPGC-Ni determines the distribution of conducting graphitized carbon and quaternary N in HPGC. SEM images of HPGC-Ni and the corresponding EDS mapping images are displayed in Figure 2 to investigate the distribution of Ni. HPGC-Ni was formed through the process of carbonization, activation, and catalysis. SEM image shows that the HPGC-Ni sample exhibits an alveolate-like porous structure with a pore diameter of about 1 μm. The porous structure was generated due to the etch of KOH activation reagent through a redox reaction [26]. Besides, there are many Ni particles with a diameter of about 200 nm can be seen in the carbon matrix. Ni particles were formed by the carbothermal reduction of nickel acetate. The C, N, and O elements in EDS mapping images come from the precursor PPy. The corresponding EDS mapping images display the distribution of Ni element is consistent with that of C, N, and O elements, indicating that Ni nanoparticles are uniformly encapsulated into the carbon frameworks.

**Figure 3:**
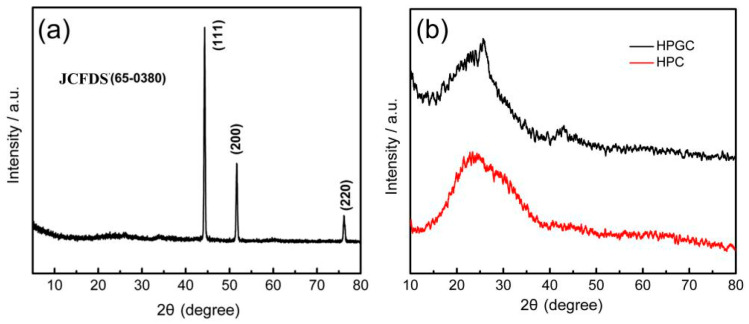
X-ray diffraction (XRD) patterns of HPGC-Ni (**a**), HPGC and HPC (**b**).

**Figure 4:**
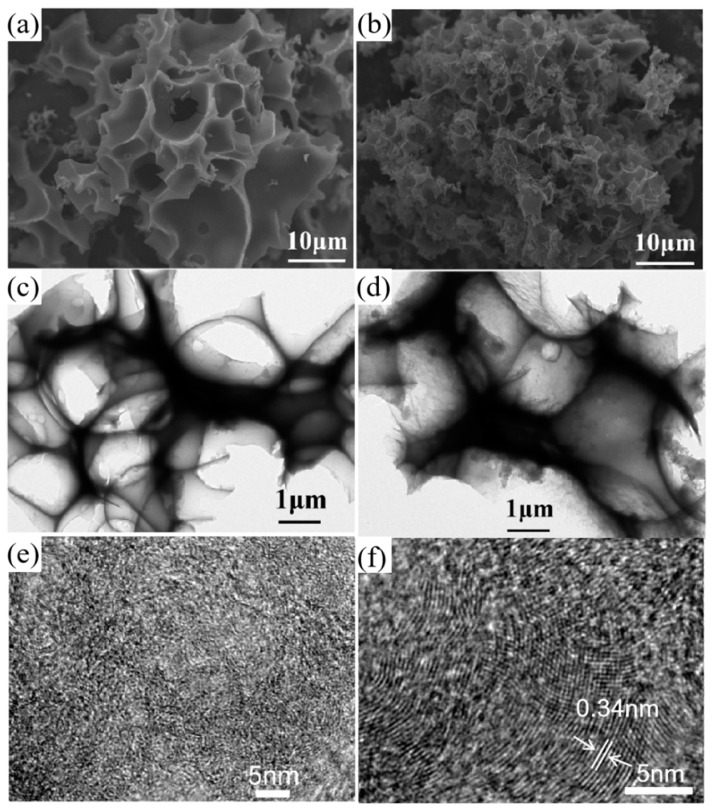
SEM (**a**,**b**), transmission electron microscopy (TEM) (**c**,**d**), and high-resolution TEM (HRTEM) (**e**,**f**) images of HPC (**a**,**c**,**e**) and HPGC (**b**,**d**,**f**) samples.

The existential form of nickel element in HPGC-Ni, and the graphitization degree of HPGC caused by nickel element were investigated by XRD patterns in Figure 3. As shown in Figure 3a, the diffraction peaks of HPGC-Ni located at 44.2°, 51.5°, and 76.1° can be indexed to the (111), (200), and (220) crystal planes of metallic Ni (Joint Committee on Powder Diffraction Standards, JCPDS card, no. 65-0380), respectively [31]. Nickel existed as an elementary substance, which agrees with the result from SEM images of HPGC-Ni. The diffraction peaks of carbon cannot be found due to the high crystallinity of nickel. XRD patterns of HPGC shows that the diffraction of peaks of Ni disappeared after the etching of HCl solution, indicating that the Ni nanoparticles were completely removed (Figure 3b). The disappearance of Ni nanoparticles is validated by the following SEM image of HPGC. Both HPGC and HPC show one broad peak at 24.7° due to amorphous carbon structures. Two peaks of HPGC at 25.6° and 42.7° correspond to (002) and (101) planes of graphitized carbon, respectively [2]. According to Bragg’s equation, the d_002_ value for HPGC material is calculated to be 0.345 nm, which is smaller than that of HPC (0.36 nm). The narrower d_002_ value implies higher crystallinity and degree of graphitization, which can also be demonstrated by HRTEM images. As shown in Figure 4e, no distinct lattice distance can be found in the HRTEM image, suggesting the amorphous state of the HPC. However, the HRTEM image of HPGC in Figure 4f shows the distinct crystal lattice distance with a large interlayer space of 0.34 nm, in line with the interplane distance of the (002) lattice of graphite layers [25]. The result indicates a highly graphitized structure of HPGC, owing to the catalytic action Ni nanoparticle [32]. The formation mechanism of graphitized structure is shown in Figure 1. At high temperature, the Ni metal crystals ripen and amorphous carbon is taken up by Ni. After that, the carbon film is dissolved and graphene layers nucleate and grow on the Ni surfaces. Finally, Ni retracts and graphitized carbon becomes visible due to the inherent instability of Ni lamella and high temperature. Increased electrical conductivity of HPGC caused by a high graphitization degree can contribute to high rate capability and energy efficiency.

The presence of nickel catalyst not only increases graphitization degree of material, but also changes morphology of material and creates more interconnected pores. To observe the morphology of HPGC, representative SEM, TEM, and HRTEM images were recorded with HPC for comparison. The SEM image of HPGC in Figure 4b exhibit a three-dimensional alveolate-like morphology with abundant open and interconnected pores. The disappearance of Ni particles in HPGC is in keeping with the result of the XRD pattern. The SEM image of HPC is similar to that of HPGC, but HPC has significantly fewer folds and holes. The formation of the pores is attributed to not only the etching effect of the KOH activation reagent, but also the retraction and disappearance of Ni. A few macropores of 2–10 μm in HPC changes to abundant macropores of <2 μm in HPGC due to the presence of nickel catalyst. Large quantities of macroporous voids can provide ion-buffering reservoirs and minimize the diffusion distance to the interior surface during the charge and discharge process. Therefore, the abundant macropores in HPGC can bring about high power density and high-rate capacitive performance. TEM images in Figure 4c,d reveal that both HPC and HPGC samples has plenty of micropores, mesopores, and macropores. However, there are more interconnected micro/nano holes in HPGC, which offered vast active sites for electrochemical reactions, plentiful interfaces for charge accumulation, and short paths for ion/electron transport.

**Figure 5:**
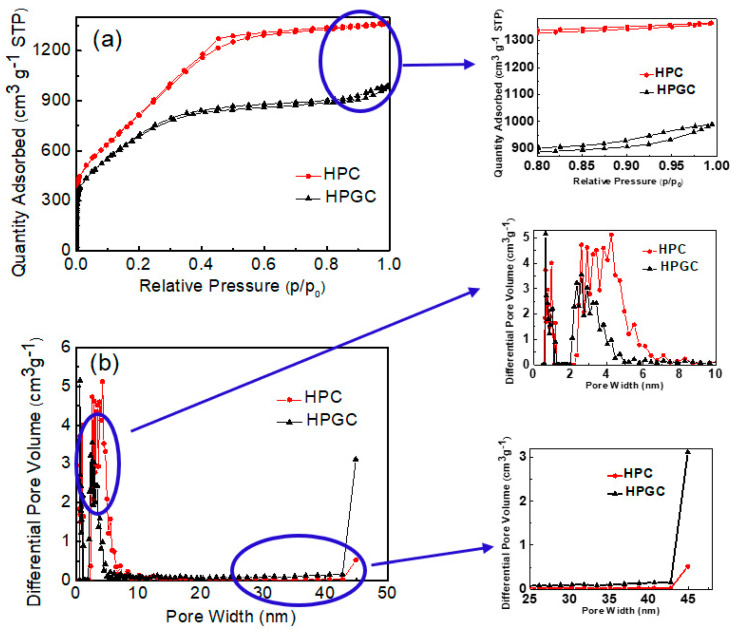
N_2_ adsorption-desorption isotherms (**a**) and pore size distribution curves (**b**) of HPC and HPGC samples (the inset figures show enlarged curves).

Pore size distribution and the specific surface area are important factors for the capacity of the electric double-layer capacitor. N_2_ adsorption-desorption isotherms were tested to further analyze the pore structures of HPC and HPGC (Figure 5a). Both HPC and HPGC present combined features of type-I isotherm and type-IV isotherm. A steep of nitrogen uptake at a low relative pressure indicates the formation of numerous micropores, while a hysteresis at a high relative pressure (P/P0 = 0.8–1.0) suggests a mesoporous material. The BET specific surface area of HPGC and HPC was calculated to be 2489.2 m^2^ g^−1^ and 3000.6 m^2^ g^−1^, respectively. The pore volume of HPGC and HPC calculated by the BJH method was 1.61 cm^3^ g^−1^ and 2.11 cm^3^ g^−1^, respectively. As shown, the larger specific surface area and pore volume of HPC is attributed to more micropores. However, the decrease of ion-transport resistance depends mostly on mesopores in porous carbons [21]. Figure 5b shows the pore size distribution curves of HPC and HPGC samples. The similar pore size distribution and BET specific surface area are determined by KOH activation. Moreover, HPC displays a relatively narrow pore size distribution of 1–7 nm. However, the pore size of HPGC centers on 1–5 nm and 25–45 nm. For HPGC, the retraction of Ni at high temperature may cause the change of pore size and lead to more mesopores of 25–45 nm. Compared with pores of 1–5 nm, the mesopores of 25–45 nm are beneficial to ions transport. The nitrogen adsorption-desorption measurements join morphology proving the interconnection of micro/nano holes in HPGC. The structure of interconnection may be due to the retraction and dissolution of Ni, which is beneficial for the charge storage and ion transfer (Figure 1). The graphitized hierarchical porous structure could facilitate fast electron and electrolyte ion transport during the charge and discharge process, thereby improving the capacitance at high current density.

**Figure 6:**
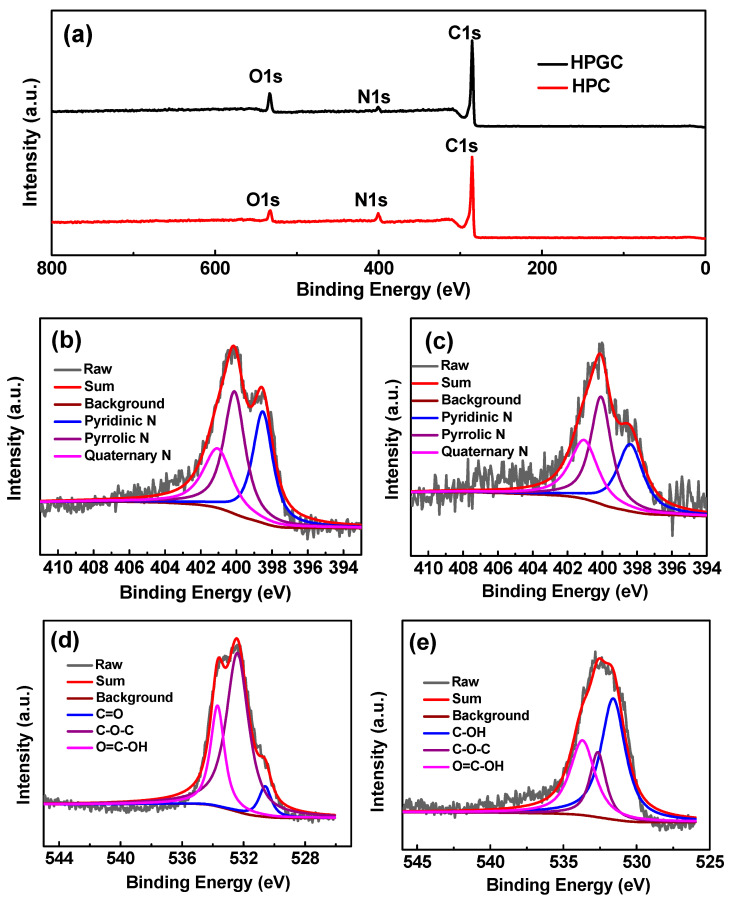
X-ray photoelectron spectroscopy (XPS) survey spectra (**a**) of the HPGC and HPC samples; High-resolution XPS spectra of N1s (**b**,**c**) and O1s (**d**,**e**) peaks of the HPGC sample (**b**,**d**) and the HPC sample (**c**,**e**).

**Figure 7:**
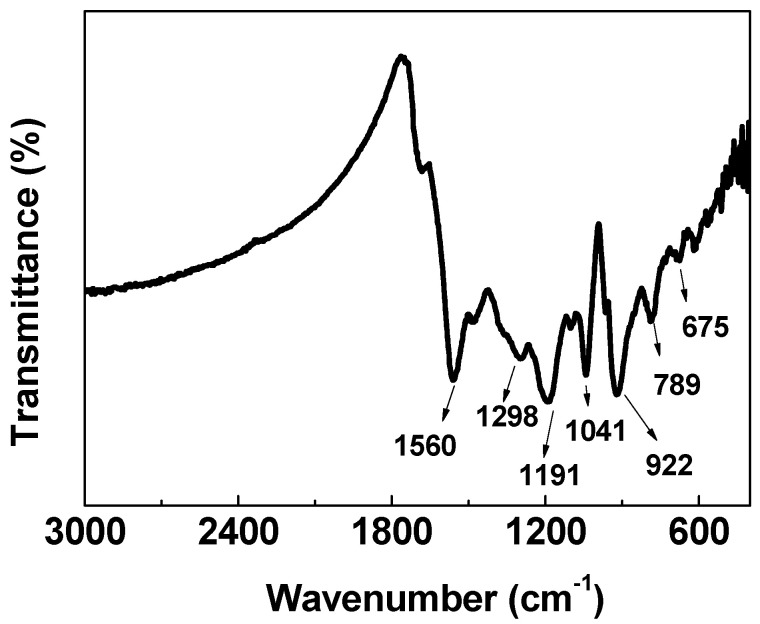
Fourier transform infrared (FTIR) spectrum of polypyrrole.

The capacitance of carbon materials can also be improved by heteroatoms. The chemical states and element content of samples were analyzed by the XPS survey spectra of HPGC and HPC (Figure 6). The atomic percentages of C, N and O calculated from XPS spectra are summarized in Table 1. It can be seen that the O content increases from 5.91 at.% for HPC to 9.11 at.% for HPGC, and the percentage of N decreases from 7.04 at.% for HPC to 3.52 at.% for HPGC. N and C elements of two samples originate from precursor PPy. The FTIR spectrum of PPy (Figure 7) presents the characteristic peaks at 1560 (C-C stretching vibration), 1298 (C–N in-plane deformation), 1191 (C–C bending vibration), 1041 (C–H in-plane deformation), 922 (C–H out-of-plane ring deformation), 789 (C–H) and 675 cm^−1^ (C–C out-of-plane ring deformation) [33]. The increase of the O content is attributed to the incorporation of oxygen in nickel acetate during the carbonization-activation process. HPGC and HPC two carbon materials have almost the similar total content of heteroatoms. The high-resolution N 1s spectrum can be resolved into three individual peaks corresponding to pyridinic N (398.4 ± 0.3 eV), pyrrolic N (400.3 ± 0.3 eV), and quaternary N (401.1 ± 0.3 eV), respectively (Figure 6b,c) [16]. Compared with HPC, HPGC had higher quaternary N content which was 29.29 at.% (Table 1). It can be concluded that the presence of Ni catalyst is beneficial to the formation of quaternary N during the carbonization process of the PPy precursor. Quaternary N possesses strong ability of giving electrons, which can effectively enhance the conductivity of the material and improve capacity retention at high rates. Furthermore, pyrrolic N and pyridinic N can produce additional pseudocapacitance, while quaternary N can enhance the ability of generating pseudocapacitance. The existence of surface oxygen functional groups is attributed to the incorporation of oxygen from potassium hydroxide and nickel acetate. For HPGC in Figure 6d, the high-resolution

O1s spectrum can be fitted into three peaks corresponding to quinone C=O (530.6 ± 0.3 eV), C–O–C (532.9 ± 0.3 eV), and O=C–OH groups (533.7 ± 0.3 eV). However, the high-resolution O 1s spectrum of HPC exhibits phenol C–OH (531.8 ± 0.3 eV), C–O–C, and O=C–OH groups (Figure 6e) [19]. Among these oxygen functional groups, only quinone C=O in HPGC is able to generate additional faradic pseudocapacitance. Therefore, nickel acetate catalyst can promote the formation of quaternary N and quinone C=O to enhance the conductivity of the material and to generate additional pseudocapacitance. The HPGC can be expected to have higher capacitance and capacity retention than HPC.

**Figure 8:**
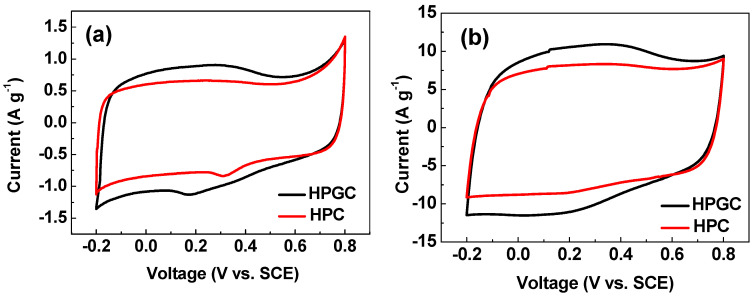

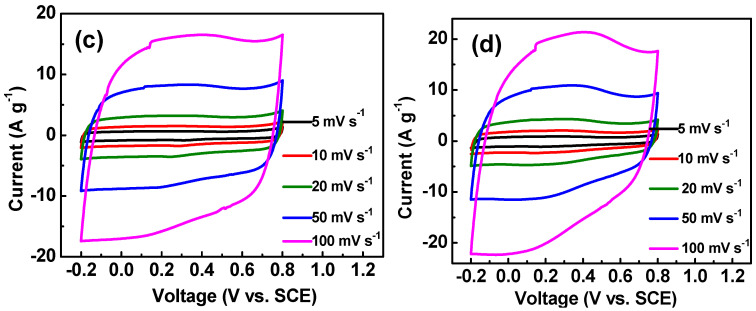
Cyclic voltammetry (CV) curves of HPGC and HPC electrodes at scan rates of 5 mV s^−1^ (**a**) and 50 mV s^−1^ (**b**); CV curves of HPC (**c**) and HPGC (**d**) electrodes at different scan rates from 5 mV s^−1^ to 100 mV s^−1^.

In order to evaluate the electrochemical capacitive properties of HPGC, CV curves were performed in 1M H_2_SO_4_ aqueous electrolyte. Figure 8a displays the CV curves of HPC and HPGC electrodes at a scan rate of 5 mV s^−1^. Both HPC and HPGC present similar CV curves which are rectangular-like shape with humps. This is a combined result of electric double layer formation and pseudocapacitive contribution produced by the redox reaction of functional groups, including pyrrolic N, quaternary N, and quinone C=O. The possible redox reactions show below [22].

>C=NH + 2 e^−^ + 2 H^+^ ↔ >CH–NH_2_ (1)

>C–NHOH + 2 e^−^ + 2 H^+^ ↔ >C–NH_2_ + H_2_O (2)

>C=O + H^+^ + e^−^ ↔ >C–OH (carbonyl, basic) (3)

>C=O + e^−^ ↔ >C–O^−^ (quinone, basic) (4)

Compared with the HPC electrode, the HPGC electrode possesses a larger enclosed area at various scan rates, indicating a higher specific capacitance (Figure 8a,b). Figure 8c,d show the CV curves of two samples at scan rates from 5 to 100 mV s^−1^ in a voltage of −0.2–0.8 V. With the increase of scanning rates, the CV curves of both the HPC and HPGC electrodes maintain a quasi-rectangular shape, implying the good rate performance of HPGC and HPC. The good rate performance can be attributed to the interconnected micro/nano holes that facilitate fast electrolyte transfer.

**Figure 9:**
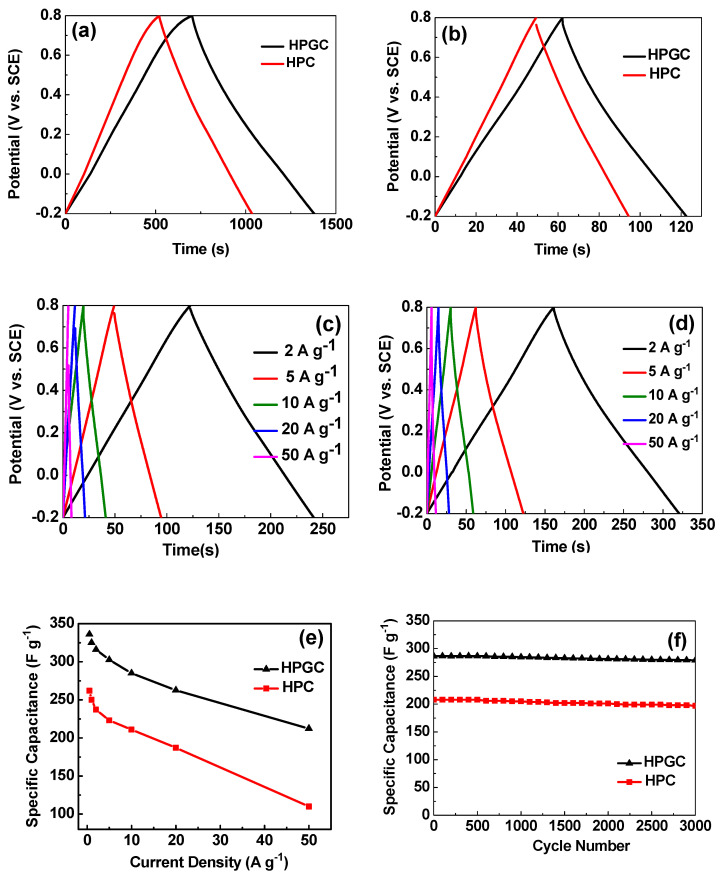
The galvanostatic charge and discharge profiles of the HPGC electrode and HPC electrode at current densities of 0.5 A g^−1^ (**a**) and 5 A g^−1^ (**b**); The galvanostatic charge and discharge profiles of the HPC (**c**) and HPGC (**d**) electrodes at various current densities. (**e**) The specific capacitance of the HPC and HPGC electrodes at different current densities; (**f**) Cycling tests of the HPGC and HPC electrodes at a current density of 10 A g^−1^ for 3000 cycles.

Galvanostatic charge-discharge (GCD) measurement is another way to value capacitive properties. Figure 9a,b display the GCD curves for two electrodes at current densities of 0.5 and 5 A g^−1^, respectively. The GCD curves of HPGC and HPC electrodes are not strictly symmetrical. The little deviation from linear property is attributed to the pseudocapacitance effect resulted from functional groups. The deviation in the curve of the HPGC electrode is larger than that of the HPC electrode, suggesting a greater contribution of pseudocapacitance. This is consistent with the result of XPS analysis. Besides, the charge-discharge time of the HPGC electrode is significantly longer than that of the HPC electrode, indicating a higher specific capacitance of HPGC electrode [16]. The result is in good agreement with that obtained from CV curves. Figure 9c,d show the GCD curves of both the HPGC electrode and HPC electrode at various current densities. Both HPGC and HPC display nearly symmetrical curves without obvious voltage drop even at a current density of 50 A g^−1^, suggesting good reversibility for charge storage and transfer. The rate performances of electrodes are reflected in Figure 9e. The HPGC shows much higher specific capacitance values at various current densities, in comparison with the HPC electrode. HPGC electrode delivers capacitance of 336.3 F g^−1^ at 0.5 A g^−1^, which is much higher than that of the HPC electrode (262.2 F g^−1^). The capacitance of the HPGC electrode is 26% higher than that of the HPC electrode, when the current density is 10 A g^−1^. Furthermore, the as-prepared HPGC shows a higher specific capacitance than most other oxygen- and nitrogen-enriched porous carbon materials (Table 2). Even at a high current density of 50 A g^−1^, the specific capacitance of HPGC still reaches as high as 212.2 F g^−1^. However, the HPC electrode only shows a specific capacitance of 110.0 F g^−1^ at 50 A g^−1^. The greatly improved rate capability of HPGC can be ascribed to the graphitization and abundant conjoint pores of HPGC. The limited extent of graphitization may be a major cause of capacity loss with the increase of current density. The HPGC electrode also exhibits a higher cycling stability with 97.4% capacitance retention after 3000 cycles at a current density of 10 A g^−1^ (Figure 9f). For the HPC electrode, the capacitance retention is 94.7%, which is slightly lower than that of HPGC. The remarkable cycling stability of HPGC benefits not only from excellent chemical reversibility of the pseudocapacitive nitrogen- or oxygen-containing functional groups during the charge and discharge process, but also from the enhanced electron conductivity and wettability of the electrolyte towards the electrode surface.

**Figure 10:**
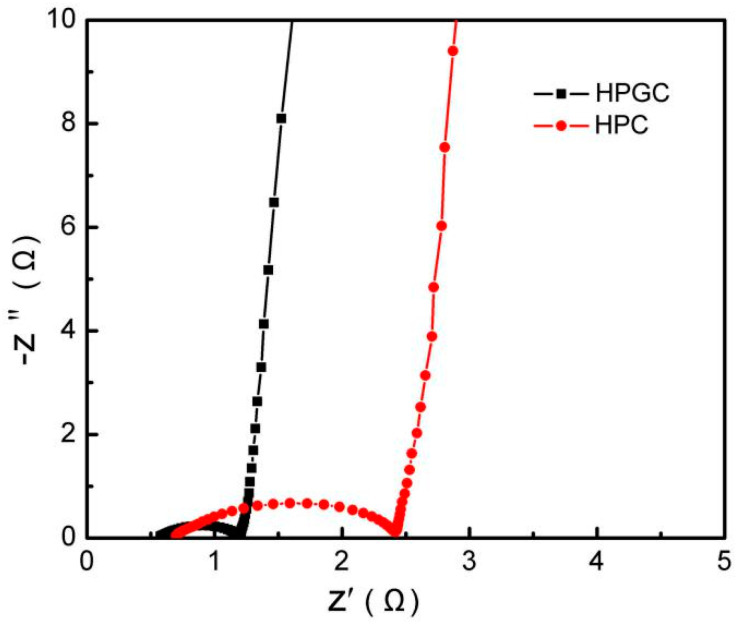
Nyquist plots of HPGC and HPC electrodes.

The Nyquist plots in Figure 10 were employed to confirm the improved kinetics for electron transport in the HPGC supercapacitor. Both HPGC and HPC electrodes display near-vertical slopes at low-frequency, indicating nearly ideal capacitive properties with fast ion diffusion. The low ion migration resistivity at the electrode/electrolyte interface is mainly caused by connected pores structure and good surface wettability by the introduction of heteroatoms. It is noticeable that HPGC has a smaller diameter of the semicircle in the high frequency region, compared with HPC. Hence, the electric resistance of HPGC is lower than that of HPC, owing to the high electronic conductivity of the HPGC electrode. Its superior electrical conductivity results from graphitized carbon and quaternary N, which were produced by carbonization with nickel acetate catalyst. As a result, the increased electrical conductivity of HPGC can lead to improved rate capability and cycling stability.

## 4. Conclusions

In conclusion, we have taken a one–step carbonization-activation-catalytic approach to fabricate novel and functionalized HPGC. It is a significant challenge to derive carbons with both a high graphitization degree and abundant ion-diffusion channels. In our work, Ni catalyst played a significant role in enhancing the electrical conductivity, introducing pseudocapacitance, and promoting ion diffusion. On the one hand, nickel catalyst could promote the formation of graphitized carbon, quaternary N and quinone C=O to enhance the conductivity of the material and to generate additional pseudocapacitance. On the other hand, the retraction and dissolution of Ni catalyst caused the change of pore size and interconnection of pores in material, obtaining plentiful interfaces for charge accumulation and short paths for ion/electron transport. Therefore, compared with HPC, HPGC shows enhanced energy-storage performances, such as enlarged specific capacity (336.3 F g^−1^ at a current density of 0.5 A g^−1^), higher rate capacity (212.2 F g^−1^ at a current density of 50 A g^−1^), and more excellent cycling stability (97.4% capacitance retention after 3000 cycles at a current density of 10 A g^−1^). Moreover, the as-prepared HPGC shows a higher specific capacitance than most other oxygen- and nitrogen-enriched porous carbon materials. HPGC is expected to be a promising electrode material for supercapacitors due to its excellent energy-storage performances and easy mass preparation.

## Figures and Tables

**Table 1:** The C, N and O contents in the HPGC and HPC samples obtained from XPS spectra.

Samples	C_XPS_ (at.%)	N_XPS_ (at.%)	O_XPS_ (at.%)	Nitrogen Contents from XPS (at.%)		
				Pyridinic N	Pyrrolic N	Quaternary N
HPC	87.05	7.04	5.91	34.37	39.48	26.15
HPGC	87.37	3.52	9.11	31.48	39.23	29.29

**Table 2:** Summary of electrochemical performance for oxygen- and nitrogen-enriched porous carbon electrode materials.

Carbon Precursor	SSA (m^2^ g^−1^)	Specific Capacitance (F g^−^^1^)	Reference
benzotriazole	1337.7	302.55 (0.5 A g^−^^1^)	[22]
kelp	1000	440 (0.5 A g^−^^1^)	[20]
potassium humate/manganous nitrate	1119	<225 (0.5 A g^−^^1^)	[21]
phenolic resin/polyvinylpyrrolidone	463	228 (0.2 A g^−^^1^)	[24]
Polypyrrole	2489.2	336.3 (0.5 A g^−^^1^)	This work
